# Stratification of candidate genes for Parkinson’s disease using weighted protein-protein interaction network analysis

**DOI:** 10.1186/s12864-018-4804-9

**Published:** 2018-06-13

**Authors:** Raffaele Ferrari, Demis A. Kia, James E. Tomkins, John Hardy, Nicholas W. Wood, Ruth C. Lovering, Patrick A. Lewis, Claudia Manzoni

**Affiliations:** 1 Department of Molecular Neuroscience, UCL Institute of Neurology, Queen Square, London, WC1B 5EH UK; 2 School of Pharmacy, University of Reading, Whiteknights, Reading, RG6 6AP UK; 3 Centre for Cardiovascular Genetics, Institute of Cardiovascular Science, University College London, London, WC1E 6JF UK

**Keywords:** Bioinformatics, Networks, Functional genomics, Protein-protein interactions, Pathways, Neurodegeneration, Parkinson’s disease, GWAS

## Abstract

**Background:**

Genome wide association studies (GWAS) have helped identify large numbers of genetic loci that significantly associate with increased risk of developing diseases. However, translating genetic knowledge into understanding of the molecular mechanisms underpinning disease (i.e. disease-specific impacted biological processes) has to date proved to be a major challenge. This is primarily due to difficulties in confidently defining candidate genes at GWAS-risk loci. The goal of this study was to better characterize candidate genes within GWAS loci using a protein interactome based approach and with Parkinson’s disease (PD) data as a test case.

**Results:**

We applied a recently developed Weighted Protein-Protein Interaction Network Analysis (WPPINA) pipeline as a means to define impacted biological processes, risk pathways and therein key functional players. We used previously established Mendelian forms of PD to identify seed proteins, and to construct a protein network for genetic Parkinson’s and carried out functional enrichment analyses. We isolated PD-specific processes indicating ‘mitochondria stressors mediated cell death’, ‘immune response and signaling’, and ‘waste disposal’ mediated through ‘autophagy’. Merging the resulting protein network with data from Parkinson’s GWAS we confirmed 10 candidate genes previously selected by pure proximity and were able to nominate 17 novel candidate genes for sporadic PD.

**Conclusions:**

With this study, we were able to better characterize the underlying genetic and functional architecture of idiopathic PD, thus validating WPPINA as a robust pipeline for the in silico genetic and functional dissection of complex disorders.

**Electronic supplementary material:**

The online version of this article (10.1186/s12864-018-4804-9) contains supplementary material, which is available to authorized users.

## Background

In the past decade, high throughput technologies, such as DNA microarray, exome and genome sequencing, have provided investigators with an extensive catalogue of genes and genetic variations associated with complex disorders [1].

Parkinson’s disease (PD) is a complex neurodegenerative condition; to date, familial PD is linked to 14 Mendelian genes (Table 1) [2], accounting for 1–5% of all cases [3], whilst for idiopathic PD (> 90% of all cases) multiple genome-wide association studies (GWAS) have identified over 30 risk loci throughout the human genome [4]. Importantly, a number of the most significant single nucleotide polymorphisms (SNPs) identified by the PD GWA studies map either directly or in close proximity to previously identified Mendelian genes (e.g. *LRRK2*, *SNCA* and *GBA*) indicating that such genes likely contribute to disease over a continuum of strong (Mendelian forms) and small to moderate (GWAS hits) effect size [5]. Yet, for the majority of the PD genome wide risk loci (and generally in GWAS), there is currently no consensus on how to select candidate genes in association with the most significant risk SNP(s) [6].

**Table 1:**
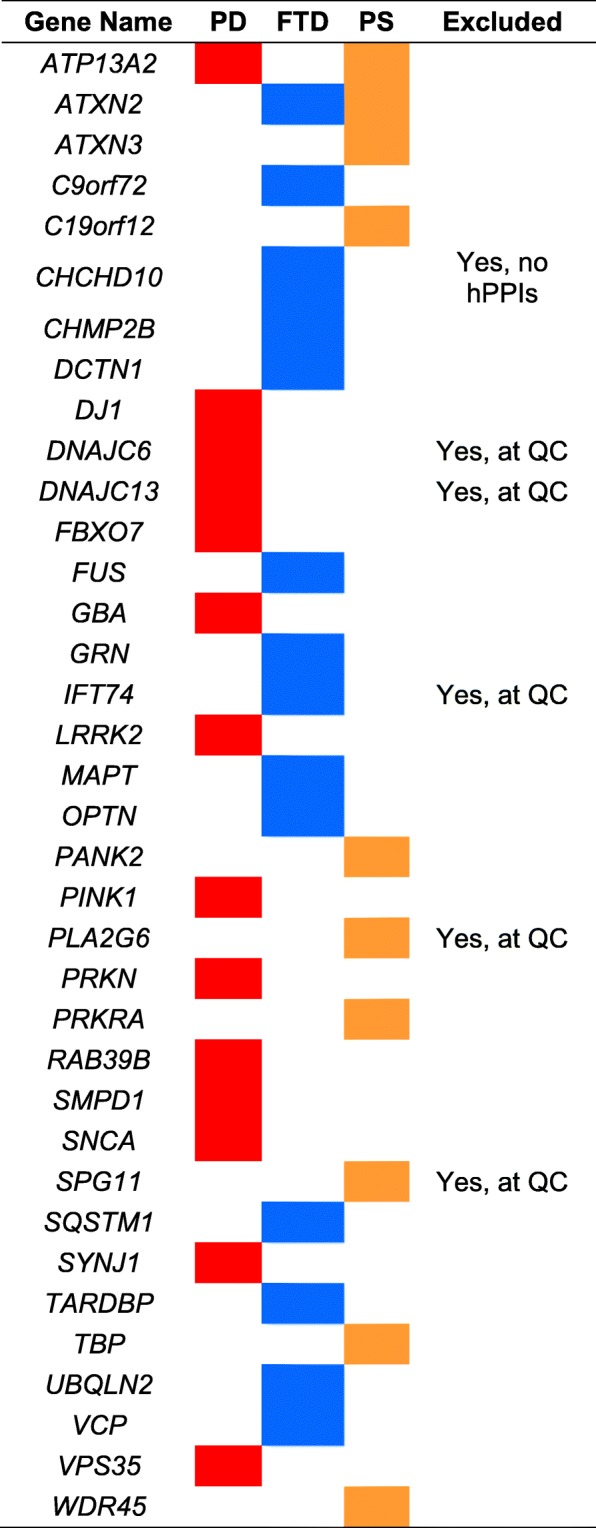
List of seeds

*PD* Parkinson’s Disease, *FTD* frontotemporal dementia, *PS* parkinsonian syndromes

Interpreting GWAS results is a challenging task [6, 7] as not only is it difficult to confidently define the authentic associated marker (the top identified SNP might be in linkage disequilibrium [LD] with the real associated SNP), but also it is now recognized that the practice of choosing candidate genes by proximity to a strongly associated SNP is unlikely accurate [8–10]. Furthermore, there are significant challenges in then characterising functional/biological effects to the top SNPs. This clearly indicates that making the step from genomic insight(s) to functional translation is currently arduous due to the limited success in progressing from genomic loci to candidate genes, coupled with the prevalent functional practice of studying one gene at a time.

In this study, we applied a systematic computational approach to investigate the interactome of the PD-Mendelian genes through weighted protein-protein interaction network analysis (WPPINA) [11] as a means to define PD-specific impacted pathways and stratify candidate genes within PD-GWAS loci. Specifically, we selected proteins encoded by PD-Mendelian genes as seeds, and harvested data of wet-lab proven protein-protein interactors (PPI) to build the PD-interactome for which we then performed functional annotation analysis to highlight impacted biological processes and to list all the proteins contributing to these systems. Finally, we mapped the genes encoding all such proteins to the loci highlighted in the PD meta-analysis [4] to assist identification of candidate genes driving risk in sporadic PD on the basis of available functional knowledge.

Using PD as a case study, we demonstrate that WPPINA, in combination with genomic approaches, is a powerful and adaptable tool to assess and prioritize candidate genes within risk-loci of complex disorders.

## Results

### Design

We sought to consider the interactomes associated with the parkinsonian syndromes, which manifest in classical PD - the focus of the current study – and a subset of cases diagnosed with frontotemporal dementia (FTD) with parkinsonism and in a group of conditions classified under the umbrella term ‘parkinsonian spectrum’ (PS, see also Additional files 1 and 2: Table S1). We used all known Mendelian genes associated with these conditions as seeds (Table 1) to build syndrome-specific networks to: i) highlight syndrome-specific impacted biological processes and relevant nodes therein, and; ii) aid prioritization of candidate genes within the PD-GWAS loci. In this fashion, we were able to test for specificity of our method prior to carrying forward the PD-GWAS candidate genes prioritization on the basis of the PD-specific impacted biological processes and the key proteins therein.

### Network construction and topological analysis

For each Mendelian gene used as a seed the WPPINA builds a protein network composed of the seeds, their direct interactors (first layer nodes) and the direct interactors of each first layer node (second layer nodes).

**Fig. 1:**
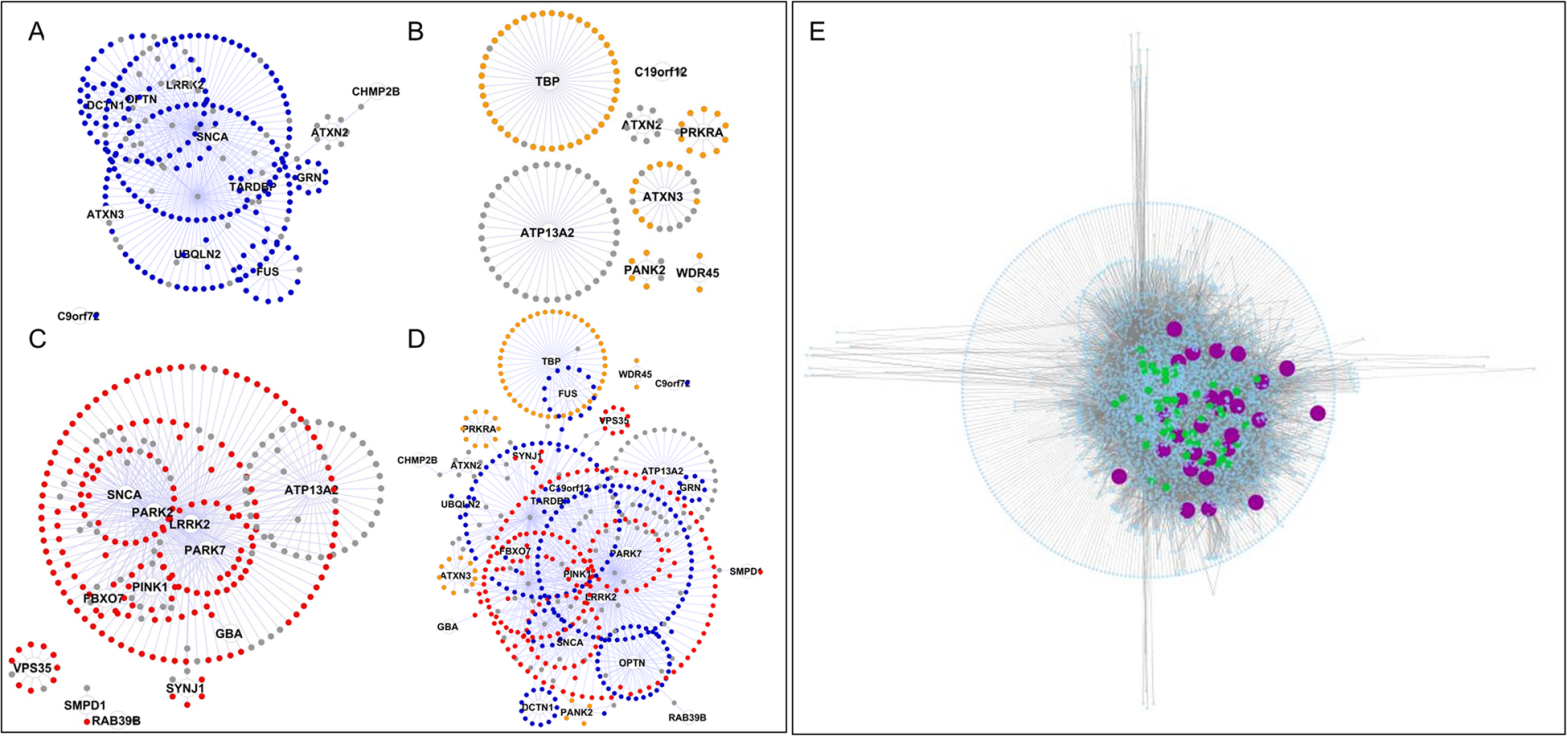
First layer protein networks. **a**. FTD (blue nodes, 272 nodes and 304 edges); **b**. PS (orange nodes, 140 nodes and 133 edges); **c**. PD (red nodes, 286 nodes and 332 edges); **d**. Combination of **a**-**b**-**c** into a single network: the grey nodes represent proteins that are shared between **a**-**b**, **a**-**c**, **b**-**c** or **a**-**b**-**c**. Networks are reported as organic layout to highlight clustering properties. **e**. Seeds + first and second layer interactors combined in the final network, purple = seeds; green = IIHs

The 3 individual first-layer-networks (PD, FTD and PS) are shown in Fig. 1a-c. The combination of the 3 networks generated a nearly completely connected first-layer-network (Fig. 1d). Due to the seed centric approach used to construct the network, the first layer of a network is partially dependent upon hypothesis driven experiments. Moreover, the interactomes are subject to ascertainment bias resulting in interactomes of different sizes [12]. The ascertainment bias is an intrinsic property of the PPI networks, yet the seed centrality bias can be diluted by scaling up the network to the second layer, therefore including nodes that were not necessarily studied as direct interactor(s) of the seeds under investigation [11].

The construction of the second layer resulted in a comprehensive network made of 5414 nodes and 18,492 undirected edges (Fig. 1e); the seed-centrality issue was diluted as confirmed by an increase in average number of neighbors and connection density, and by a decrease of the average shortest path (Additional file 3). The analysis of the first + second layer networks led to the identification of inter-interactome hubs (IIHs) that are nodes with a degree of interactome connection (IDC) ≥ 0.6 (Additional file 4). These nodes represent the shortest path to connect more than 60% of the seed genes, therefore (based on the network parsimony hypothesis [13]) it is reasonable to expect these to reside in disease relevant pathways, congruent with the majority of the seeds, and therefore at the core of the syndrome-specific network.

### Network functional analysis

We first analyzed the entire network (input of 5414 nodes) for enrichment of Gene Ontology (GO) biological processes (BPs) terms (Additional file 5); we then applied the same procedure to the IIHs for PD, FTD and PS separately (Additional files 6, 7 and 8) [12] highlighting a subset of semantic classes indicative of syndrome-specific biological processes (Additional file 9).

**Fig. 2:**
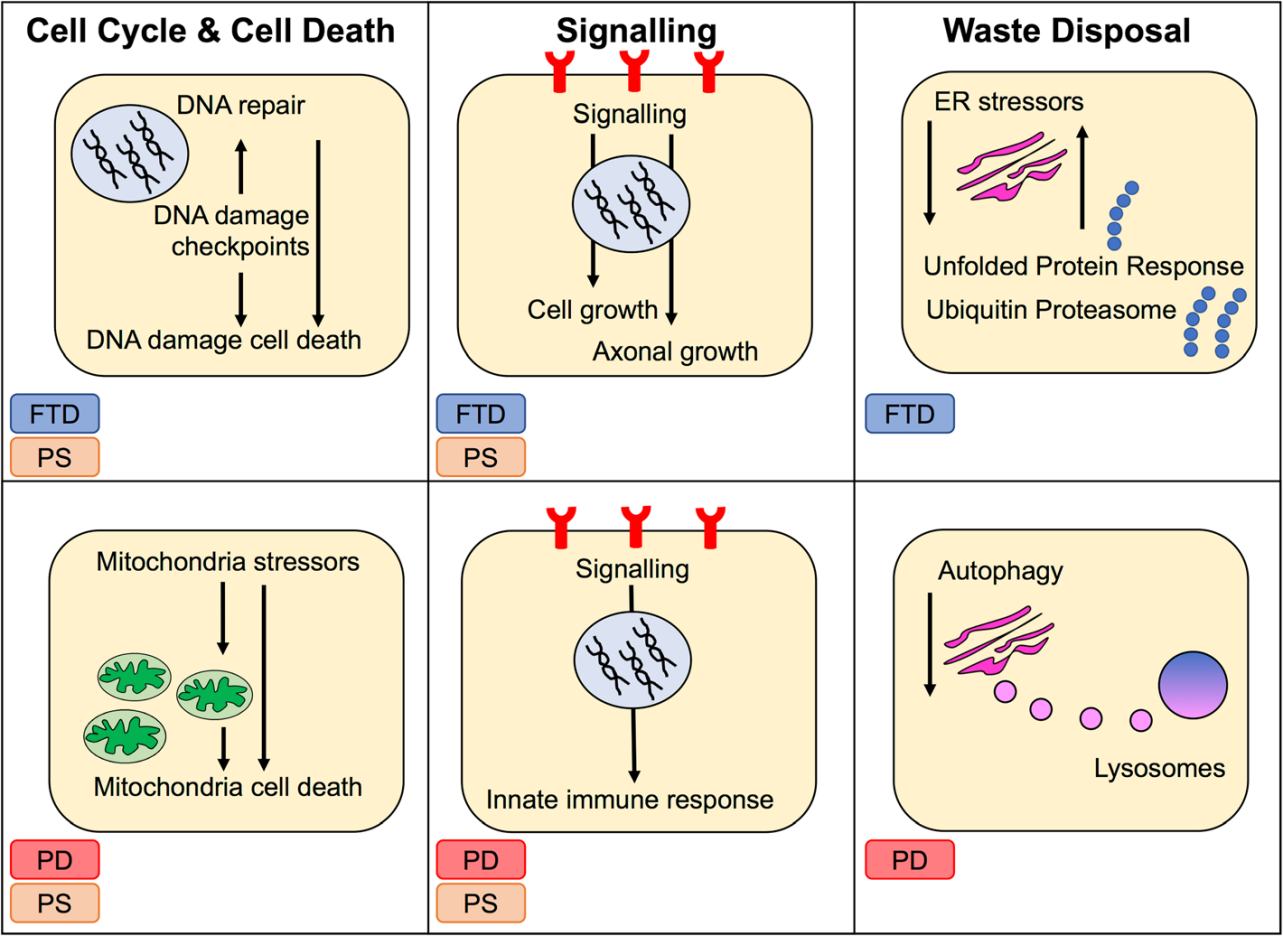
Most impacted biological processes. Three major impacted functional blocks – ‘cell cycle & cell death’, ‘signalling’ and ‘waste disposal’ – differently segregated across the different syndromes (PD: red, FTD: blue and PS: orange)

Although ‘RNA metabolism’, ‘organelles’, ‘adhesion’, ‘cytoskeleton’ and ‘chromatin’ are general terms, a number of functional blocks clearly supported syndrome-specific processes (Fig. 2 and Additional file 10) cell cycle and DNA metabolism (semantic classes: ‘cell cycle checkpoints’, ‘DNA damage check point’ and ‘repair’) were specific to FTD/PS. Cell death pointed to ‘intrinsic apoptosis‘ for both FTD/PD indicating ‘DNA damage response’ as specific to FTD, and ‘mitochondria stressors’ as specific to PD/PS. Stress indicated ‘oxidative stress’ as a common semantic class across all syndromes: ‘ER stress’ was shared across FTD and PD – complexes that activate ‘DNA damage response’ were however (again) unique for FTD – whilst ‘cell death via mitochondria’ was unique for PD. These very links were further supported by localization that pointed at the ‘nucleus’ for FTD/PS and the ‘mitochondria’ for PS/PD. Development suggested ‘cell growth’ for FTD and ‘neuronal/axonal development’ for PS, this being in line with semantic classes indicating ‘growth factors’ within signaling, that were specific for FTD and PS. Conversely, the indication of ‘cytokines’, ‘immune receptors’, ‘innate immune system’ as specific to PS and PD in the immune system functional block was supported by a plethora of immune signaling semantic classes in the signaling functional block for PS/PD (Additional file 10). Finally, waste disposal was enriched in all 3 conditions; however, FTD was specifically characterized by semantic classes such as the ‘ubiquitin proteasome system’ and the ‘unfolded protein response’, whilst PD by ‘autophagy’. Functional annotation analysis performed through Panther replicated 95.5% of the g:Profiler findings (Additional file 11).

### PD-GWAS candidate genes prioritization

**Fig. 3:**
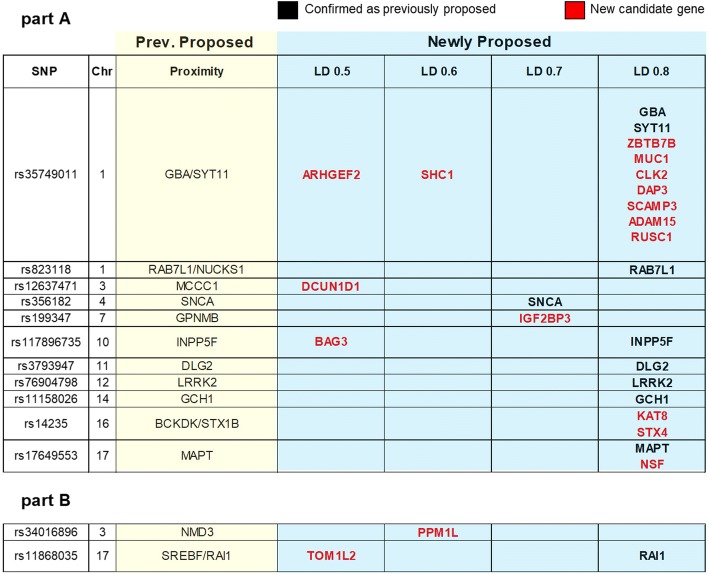
PD-GWAS gene prioritization. Significant SNPs from PD-GWAS and ORFs in LD (from r^2^ > 0.5 to r^2^ > 0.8) are shown. Part A contains significant SNPs as *per* joint analysis; part B contains significant SNPs as *per* discovery phase. The candidate genes based on proximity are summarized in the column with light yellow background. Newly proposed candidate genes identified on the basis of our analysis of the functionally relevant proteins in the PPI network are summarized in the column with light blue background. Here genes in black font confirm previous assignments by proximity, whilst genes in red font are the novel candidate genes for sporadic PD

We selected the functional blocks defined by PD-specific IIHs (Additional file 12), as they indicate conserved functions across all the Mendelian forms of PD, and extracted the list of proteins contributing to them in the complete PD-specific network (Additional file 13). We then used the significant PD-SNPs (*n* = 32) from the GWAS to define *cis*-haplotypes (Additional file 14) and map ORFs therein. Finally, we compared the ORFs with the proteins contributing to the enrichment of the PD-specific functional blocks: 19 genes were in *cis*-haplotypes defined by LD r^2^ ≥ 0.8, 2 genes in LD r^2^ ≥ 0.7, 2 genes in LD r^2^ ≥ 0.6, and 4 genes in LD r^2^ ≥ 0.5 with the top SNP of a risk locus, respectively, leading to the prioritization of a total of 27 candidate genes within the 32 PD-loci (Fig. 3).

Ten/27 candidate genes (5 of which are also Mendelian) – *GBA*, *SYT11*, *SNCA*, *RAB7L1*(*RAB29*), *INPP5F*, *DLG2, LRRK2*, *GCH1*, *MAPT*, and *RAI1* – previously selected by proximity were confirmed by our functional approach. The remaining 17/27 candidate genes – *ZBTB7B*, *MUC1*, *CLK2*, *DAP3*, *SCAMP3*, *ADAM15*, *RUSC1*, *SHC1*, *ARHGEF2*, *KAT8*, *STX4*, *IGF2BP3*, *PPM1L*, *DCUN1D1*, *NSF*, *BAG3* and *TOM1L2* – are to be regarded as novel PD candidate genes. Cell type specific expression for each prioritized candidate gene was calculated from the dataset generated by Zhang et al. [14] showing that neurons and mature astrocytes almost equally share expression of the prioritized candidate genes (Additional files 15 and 16).

To validate this analysis, we ran 100,000 random simulations. The *p*-values associated with the experimental analysis in comparison with the random distribution showed strong statistical significance (Additional file 17, *p* = 0.004 for LD r^2^ ≥ 0.5 and *p* = 0.005 for LD r^2^ ≥ 0.8). Analytic *p*-values values generated using the hypergeometric distribution also led to statistically significant results (*p* = 0.009 for LD r^2^ ≥ 0.5 and *p* = 0.012 for LD r^2^ ≥ 0.8). We undertook an additional validation step by assessing the total number of annotations reported in GO for each ORF within the analyzed haplotypes to verify potential annotation bias possibly impacting the specificity of the GWAS loci prioritization. As shown in Additional file 18, the number of annotations per genes in GO does not affect prioritization specificity.

## Discussion

Familial and sporadic PD cases associate with highly/moderately penetrant variants in a number of Mendelian genes and multiple risk loci with small to moderate effect size, respectively. Despite an abundance of genetic data, translating PD genetics into understanding of the functional and molecular mechanisms underlying PD pathogenesis is a challenging task. For Mendelian genes, this is partially due to differences in the pace of genetic data generation (fast) versus functional and molecular assessments (slow) that still evaluate one gene at a time. For sporadic cases, their association with multiple risk markers (that indicate loci rather than genes) results in additional theoretical and practical issues affecting the design of functional experiments.

A number of methods to prioritize genes within GWAS loci have recently emerged: burden scoring at gene or pathway level [15, 16], GWAS data integration with cis-eQTL signals or epigenetic markers [17–20], and PPIs-based methods [9, 21]. However, while these hold much promise, none has yet solved the issue of confidently prioritizing genes within GWAS loci and it is still the case that in published GWAS nominated genes are assigned to the top SNPs predominantly by proximity, an approach that not only is arbitrary but also inaccurate and ineffective to gather insight on molecular mechanisms impacted in disease pathogenesis [8]. Therefore, there is an urgent requirement for additional pipelines to further and better characterize GWAS loci as well as the impacted biological processes, risk pathways and therein key functional players for potential future targeting is real [1, 22].

In this view, our recently developed pipeline, WPPINA [11], is complementary to existing models given that it builds protein networks as a direct function of the disease under investigation (i.e. on the basis of their Mendelian genetics). By applying this pipeline to PD, FTD and PS we were able to highlight syndrome-specific PPI networks, impacted biological processes and associated functional players at the core of each disease (Fig. 2). We found that specific processes were differentially relevant across the 3 syndromes: ‘immune response’, particularly ‘signaling in immune response’, was relevant for PD and PS, ‘DNA damage response’ was relevant for FTD only, and ‘waste disposal’ was differentially relevant for PD (i.e. ‘autophagy’) and FTD (i.e. ‘unfolded protein response’ and ‘ubiquitin proteasome system’). Particularly, we isolated ‘mitochondria stressors’ and ‘mitochondria mediated cell death’, ‘immune response and signaling’ (i.e. ‘immune system receptors’ and ‘cytokine signaling’), and ‘waste disposal’ mediated through ‘autophagy’ as PD-specific processes.

After verifying the suitability of our approach in highlighting syndrome-specific impacted biological processes, we then focused on the PD-interactome to prioritize candidate genes within PD-GWAS loci (Fig. 3) [4] on the basis of functional relevance rather than proximity. We used the totality of the proteins indicated by the PD-specific processes and mapped them onto the PD-GWAS loci, confirming 10 candidate genes (previously nominated by proximity) and identifying 17 novel candidate genes (Fig. 3 and Additional file 15) for sporadic PD. Of note, for some of the loci we identified more than one candidate gene. This could be due to a number of reasons: i) the top SNP at the risk locus may mask a lower (but still biologically significant) signal of a second, independent SNP [23], and/or; ii) the same SNP(s) might influence more than one ORF [24] as, for example, in the case of rs34311866 for which classical wet-lab approaches prioritized different genes (i.e. *GAK* and *TMEM175*) [25, 26]. It is also noteworthy that we were not able to assign a gene to every locus. This might reflect that: i) the PPI network is incomplete (i.e. partly because not all experimental data has been captured by manual curation, and partly because the network can only be as complete as the experiments that have been performed and published), and/or; ii) we may have lowered the number of potentially positive nodes by using a stringent filtering to only retain robustly validated interactors in the network. Finally, the risk loci may target functional elements that reside in -*trans* rather than in -*cis* (e.g. distal enhancers or silencers) or non-coding RNAs, rather than protein coding genes [6].

## Conclusions

Through this in silico study of a multifactorial, complex neurodegenerative disorder, we show that our pipeline can be applied to: i) define disease-specific biological processes on the basis of known Mendelian genes, and; ii) provide a list of proteins involved in disease-specific processes that can be used to prioritize candidate genes in GWAS loci.

This pipeline aids in expanding on the genetic and functional architecture underlying idiopathic forms of disease. In doing so, it provides the basis for further genetic (e.g. such list of proteins/genes might be screened for rare variants within whole exome sequencing data sets) and hypothesis-driven functional studies to validate-risk pathways as well as identify targets for the development of therapies in the future.

## Methods

Methods are described in detail in the Additional file 1.

### Construction of the PPI network

PPIs of Mendelian-PD gene products were downloaded for each seed protein as MITAB 2.5 files (January-2016) from the IntAct [27], BioGRID [28], InnateDB, InnateDB-all, InnateDB-IMEx [29] and MINT [30] databases by means of the PSICQUIC platform (http://www.ebi.ac.uk/Tools/webservices/psicquic/view/main.xhtml) developed by the IMEx consortium [31]. All PPIs underwent quality control (QC) and filtering. Only human interaction and experimentally proven physical interaction were retained (predicted interactions and expanded complexes were removed). The interactions were then scored and thresholded taking into consideration the number of different publications and methods reporting the interaction (see [11] and Additional file 1 for details). Polyubiquitin-C, B and D (UBC, UBB and UBD) were excluded from the network as they may indicate unspecific binding of ubiquitin to proteins tagged for degradation.

### Topological analysis

The inter-interactome degree (IID) for each single node was calculated based on the number of different interactomes that node belonged to. The interactome connection degree (ICD) of a node equates to the IID divided by the total number of seeds in the network and ranges between 1 (nodes able to bridge all the interactomes in the network) and 1/number of seeds (nodes unable to bridge any interactomes in the network). Nodes with IDC ≥ 0.6 are inter-interactomes hubs (IIHs).

### Functional enrichment analysis and replication

We performed Gene Ontology (GO) [32] terms enrichment analyses in g:Profiler (g:GOSt, http://biit.cs.ut.ee/gprofiler/) [33] (October–November 2016) and replicated in PANTHER (June 2017). Enriched GO-BP terms were grouped into custom-made ‘semantic classes’; similar semantic classes were grouped into hierarchical groups called ‘functional blocks’ (Additional file 1).

### Gene prioritization – GWAS

We used thirty-two relevant SNPs as *per* the PD meta-analysis (form discovery phase and/or joint analysis) [4]. The genes mapping to the GWAS-loci (SNPSNAP, reference EU 1000G; locus definition by linkage disequilibrium (LD) r^2^ > 0.5) were matched with those encoding proteins contributing to the PD-specific risk-processes highlighted by WPPINA to aid prioritization of genes within the PD-GWAS loci. Results were statistically validated by generating 100,000 random gene-sets of sizes similar to the lists of open reading frames (ORFs) in LD with the PD-GWAS SNPs. *P*-values were calculated considering the total number of random matches falling above the actual number of experimental matches divided by the total number of trials. Analytic *p*-values were also calculated by using the hypergeometric distribution. An additional assessment was performed by evaluating the number of annotations for each gene reported in GO (GO annotation service http://amigo.geneontology.org/grebe) to verify whether differences in the annotation sizes might have influenced and driven gene prioritization.

### Cell type expression

We evaluated cell specific expression profile for genes prioritized in each PD locus. The individual expression FPKM data were downloaded for human temporal lobe cortex mature astrocytes, neurons, microglia, oligodendrocytes and endothelial cells from Additional file 1 of Zhang et al. [14].

### Software

Data was handled, filtered and scored through *in-house* R scripts (https://www.r-project.org/) as described before [11]. The final network was visualized through the freely available Cytoscape 2.8.2 software and analyzed through the network analysis plug-in [34].

## Additional files

Additional file 1: Supplementary Methods. (DOCX 64 kb)

Additional file 2: Seeds and Parkinsonian syndromes. (JPG 266 kb)

Additional file 3: Random Distribution. Distribution of the number of matches obtained in 100,000 simulated experiments in which we matched the relevant PD, process-specific, network proteins to randomly generated gene-sets of the same length as the list of ORFs in LD blocks with the top SNPS in the PD-GWAS. The distribution in blue is generated for random gene-sets of the same length as the list of ORFs in LD r^2^ ≥ 0.5; the distribution in red is generated for random gene-sets of the same length as the list of ORFs in LD r^2^ ≥ 0.8. (PDF 36 kb)

Additional file 4: Features of the PD, FTD and PS networks. A. Summary of the main features of each interactome and level of overlap across interactomes and inter-interactome hubs (IIH). B. IDC, degree of interactome connection. (PDF 259 kb)

Additional file 5: g:Pprofiler enrichment for the entire network. (XLSX 172 kb)

Additional file 6: g:Pprofiler enrichment for the FTD IIHs. (XLSX 23 kb)

Additional file 7: g:Pprofiler enrichment for the PS IIHs. (XLSX 29 kb)

Additional file 8: g:Pprofiler enrichment for the PD IIHs. (XLSX 25 kb)

Additional file 9: Enrichment Analysis. The biological process-Gene Ontology (BP-GO) terms obtained from the functional enrichment analysis of the entire network (Fig. 1d) were first grouped into semantic classes by semantic similarity and then into functional blocks. More general terms within the same functional blocks are highlighted in grey. Enrichment analysis was then performed for the PD, FTD and PS IIHs separately: enriched semantic classes from these analyses are highlighted in blue for FTD, orange for PS and red for PD. (JPG 790 kb)

Additional file 10: Functional blocks supporting syndrome-specific coherent patterns for PD, FTD and PS. Each semantic class is represented at the bottom of the x axis. Each functional block is found at the top of the x axis. The contribution of each syndrome to each semantic class is defined by colour-coded dots representing single GO terms within each semantic class (PD: red dots, FTD blue dots and PS: orange dots). The pie charts represent the relative distribution of different semantic classes *per* functional block for either syndrome. (JPG 228 kb)

Additional file 11: Panther enrichment for the entire network. (XLSX 117 kb)

Additional file 12: List of GO BPs extracted from the g:Profiler enrichment of the entire network and relevant to PD. (XLSX 103 kb)

Additional file 13: List of PD network proteins responsible for the enrichment of the terms in Supplementary File 6. (XLSX 62 kb)

Additional file 14: List of ORFs in LD with PD-GWAS top SNPs. (XLSX 15 kb)

Additional file 15: PD-GWAS gene prioritization. Significant SNPs from PD-GWAS and the number of ORFs in LD (from r2 > 0.5 to r2 > 0.8) are shown. Part A contains significant SNPs as per joint analysis, part B contains significant SNPs as per discovery phase. The candidate genes based on proximity are summarized as suggested in the original GWAS. Newly proposed candidate genes within each are identified on the basis of our analysis of the functionally relevant proteins in the PPI network. Genes previously selected by proximity and now also confirmed by functional analysis of the PD-network are in bold font. The top pie chart represents the distribution of proteins across the different relevant processes. In the final column the cell type with major expression (> 5% of average expression) is reported as calculated from the dataset generated by Zhang et al. [13] (A = mature astrocytes, N = neurons, M = microglia, O = oligodendrocytes and, E = endothelial cells). The bottom pie chart represents the distribution of proteins based on cell type expression in human temporal lobe cortex. (JPG 349 kb)

Additional file 16: Functions of newly prioritized candidate genes. (XLSX 15 kb)

Additional file 17: .GO Annotation Frequency. Proteins whose ORF is in the LD blocks around the prioritized SNPs in the PD-GWAS have been evaluate in terms of numbers of GO annotations present in GO for that specific ORF. In red are reported the proteins that correspond to genes that we prioritized with our analysis. In grey, all the other genes are reported. For some of the loci (A) the genes we prioritized was the gene with the maximum number of GO annotations for that locus; in some other cases (B) the genes we prioritized was NOT the gene with the maximum number of GO annotations for that locus. Finally, there are also mixed cases (C). (TIFF 315 kb)

Additional file 18: Second layer network topological properties. (JPG 94 kb)

## References

[1] Manzoni C, Kia DA, Vandrovcova J, Hardy J, Wood NW, Lewis PA, Ferrari R. Genome, transcriptome and proteome: the rise of omics data and their integration in biomedical sciences. Brief Bioinform. 2018;19(2):286-302. 10.1093/bib/bbw114. 10.1093/bib/bbw114 PMC6018996 27881428

[2] Hernandez DG, Reed X, Singleton AB (2016). Genetics in Parkinson disease: Mendelian versus non-Mendelian inheritance. J Neurochem.

[3] Bean LJH Stephens K, Amemiya Anne. Adam MP, Molecular Genetics. Editor-in-Chief; Senior Editors: Ardinger HH, Pagon RA, Wallace SE. Genetic Counseling. Seattle (WA): University of Washington, Seattle; 1993-2018. ISSN: 2372-0697.

[4] Nalls MA, Pankratz N, Lill CM, Do CB, Hernandez DG, Saad M, DeStefano AL, Kara E, Bras J, Sharma M (2014). Large-scale meta-analysis of genome-wide association data identifies six new risk loci for Parkinson's disease. Nat Genet.

[5] van der Brug MP, Singleton A, Gasser T, Lewis PA (2015). Parkinson’s disease: from human genetics to clinical trials. Sci Transl Med.

[6] Ward LD, Kellis M (2012). Interpreting noncoding genetic variation in complex traits and human disease. Nat Biotechnol.

[7] Furlong LI (2013). Human diseases through the lens of network biology. Trends Genet.

[8] Edwards SL, Beesley J, French JD, Dunning AM (2013). Beyond GWASs: illuminating the dark road from association to function. Am J Hum Genet.

[9] Tasan M, Musso G, Hao T, Vidal M, MacRae CA, Roth FP (2015). Selecting causal genes from genome-wide association studies via functionally coherent subnetworks. Nat Methods.

[10] Visscher PM, Wray NR, Zhang Q, Sklar P, McCarthy MI, Brown MA, Yang J (2017). 10 years of GWAS discovery: biology, function and Translation. Am J Hum Genet.

[11] Ferrari R, Lovering RC, Hardy J, Lewis PA, Manzoni C (2017). Weighted protein interaction network analysis of Frontotemporal dementia. J Proteome Res.

[12] Simoes SN, Martins DC, Pereira CA, Hashimoto RF, Brentani H (2015). NERI: network-medicine based integrative approach for disease gene prioritization by relative importance. BMC Bioinformatics.

[13] Barabasi AL, Gulbahce N, Loscalzo J (2011). Network medicine: a network-based approach to human disease. Nat Rev Genet.

[14] Zhang Y, Sloan SA, Clarke LE, Caneda C, Plaza CA, Blumenthal PD, Vogel H, Steinberg GK, Edwards MS, Li G (2016). Purification and characterization of progenitor and mature human astrocytes reveals transcriptional and functional differences with mouse. Neuron.

[15] de Leeuw CA, Mooij JM, Heskes T, Posthuma D (2015). MAGMA: generalized gene-set analysis of GWAS data. PLoS Comput Biol.

[16] Lamparter D, Marbach D, Rueedi R, Kutalik Z, Bergmann S (2016). Fast and rigorous computation of gene and Pathway scores from SNP-based summary statistics. PLoS Comput Biol.

[17] Gamazon ER, Wheeler HE, Shah KP, Mozaffari SV, Aquino-Michaels K, Carroll RJ, Eyler AE, Denny JC, Consortium GT, Nicolae DL (2015). A gene-based association method for mapping traits using reference transcriptome data. Nat Genet.

[18] Gusev A, Ko A, Shi H, Bhatia G, Chung W, Penninx BW, Jansen R, de Geus EJ, Boomsma DI, Wright FA (2016). Integrative approaches for large-scale transcriptome-wide association studies. Nat Genet.

[19] Marigorta UM, Denson LA, Hyams JS, Mondal K, Prince J, Walters TD, Griffiths A, Noe JD, Crandall WV, Rosh JR (2017). Transcriptional risk scores link GWAS to eQTLs and predict complications in Crohn's disease. Nat Genet.

[20] Zhu Z, Zhang F, Hu H, Bakshi A, Robinson MR, Powell JE, Montgomery GW, Goddard ME, Wray NR, Visscher PM (2016). Integration of summary data from GWAS and eQTL studies predicts complex trait gene targets. Nat Genet.

[21] Wang L, Matsushita T, Madireddy L, Mousavi P, Baranzini SE (2015). PINBPA: cytoscape app for network analysis of GWAS data. Bioinformatics.

[22] Hasin Y, Seldin M, Lusis A (2017). Multi-omics approaches to disease. Genome Biol.

[23] Hormozdiari F, Kostem E, Kang EY, Pasaniuc B, Eskin E (2014). Identifying causal variants at loci with multiple signals of association. Genetics.

[24] Hrdlickova B, de Almeida RC, Borek Z, Withoff S (2014). Genetic variation in the non-coding genome: involvement of micro-RNAs and long non-coding RNAs in disease. Biochim Biophys Acta.

[25] Beilina A, Rudenko IN, Kaganovich A, Civiero L, Chau H, Kalia SK, Kalia LV, Lobbestael E, Chia R, Ndukwe K (2014). Unbiased screen for interactors of leucine-rich repeat kinase 2 supports a common pathway for sporadic and familial Parkinson disease. Proc Natl Acad Sci U S A.

[26] Jinn S, Drolet RE, Cramer PE, Wong AH, Toolan DM, Gretzula CA, Voleti B, Vassileva G, Disa J, Tadin-Strapps M (2017). TMEM175 deficiency impairs lysosomal and mitochondrial function and increases alpha-synuclein aggregation. Proc Natl Acad Sci U S A.

[27] Orchard S, Ammari M, Aranda B, Breuza L, Briganti L, Broackes-Carter F, Campbell NH, Chavali G, Chen C, del-Toro N (2014). The MIntAct project--IntAct as a common curation platform for 11 molecular interaction databases. Nucleic Acids Res.

[28] Chatr-Aryamontri A, Oughtred R, Boucher L, Rust J, Chang C, Kolas NK, O'Donnell L, Oster S, Theesfeld C, Sellam A (2017). The BioGRID interaction database: 2017 update. Nucleic Acids Res.

[29] Breuer K, Foroushani AK, Laird MR, Chen C, Sribnaia A, Lo R, Winsor GL, Hancock RE, Brinkman FS, Lynn DJ (2013). InnateDB: systems biology of innate immunity and beyond--recent updates and continuing curation. Nucleic Acids Res.

[30] Chatr-aryamontri A, Ceol A, Palazzi LM, Nardelli G, Schneider MV, Castagnoli L, Cesareni G (2007). MINT: the molecular INTeraction database. Nucleic Acids Res.

[31] Orchard S, Kerrien S, Abbani S, Aranda B, Bhate J, Bidwell S, Bridge A, Briganti L, Brinkman FS, Cesareni G (2012). Protein interaction data curation: the international molecular exchange (IMEx) consortium. Nat Methods.

[32] Ashburner M, Ball CA, Blake JA, Botstein D, Butler H, Cherry JM, Davis AP, Dolinski K, Dwight SS, Eppig JT (2000). Gene ontology: tool for the unification of biology. The Gene Ontology Consortium. Nat Genet.

[33] Reimand J, Arak T, Adler P, Kolberg L, Reisberg S, Peterson H, Vilo J (2016). g:Profiler-a web server for functional interpretation of gene lists (2016 update). Nucleic Acids Res.

[34] Shannon P, Markiel A, Ozier O, Baliga NS, Wang JT, Ramage D, Amin N, Schwikowski B, Ideker T (2003). Cytoscape: a software environment for integrated models of biomolecular interaction networks. Genome Res.

