# Supplementary material for: Stratification of candidate genes for Parkinson’s disease using weighted protein-protein interaction network analysis
Source: BMC Genomics. 2018 Jun 13;19:452. doi: 10.1186/s12864-018-4804-9 (PMC6000968; doi:10.1186/s12864-018-4804-9)
Supplement: Supplementary file 1 — Supplementary Methods. (DOCX 64 kb) [file 12864_2018_4804_MOESM1_ESM.docx]

**Supplementary Methods**

**Design**

We sought to consider the broad parkinsonian syndrome, which is manifested in classical PD, the focus of the current study, as well as in a subset of cases diagnosed with frontotemporal dementia (FTD) – FTD with parkinsonism (or FTDP-17) [1] – and in a plethora of conditions such as spinocerebellar ataxia (SCA), neurodegeneration with brain iron accumulation (NBIA), spastic paraplegia (SP) and dystonia presenting with parkinsonism (DS). The latter syndromes may be classified under the umbrella term ‘parkinsonian spectrum’ (hereafter referred to as PS) [2-8]. FTD and PS were specifically selected as ‘control traits’ for PD to evaluate the power of our approach in discriminating functional processes at the basis of similar, yet different syndromes. In fact, FTD and PS not only share a number of clinical and pathological features with PD, but also are characterized by familial type of inheritance (i.e. there are syndrome-specific Mendelian genes to be used as seeds for building networks). We thus used all genes known to be associated with these conditions as seeds (Table 1) to build syndrome-specific networks.

**Definitions**

A seed is the input protein used to query and download protein-protein interactions; the interactome is the seed + its direct interactors; the first layer network is composed by the seeds and their direct interactors; the second layer network is made of all interactors of the first layer interactors; the syndrome-specific network is composed by seed + first + second layer interactomes.

**Download of the PPIs**

As detailed in [9], PPIs of Mendelian-PD gene products were downloaded for each seed protein as MITAB 2.5 files (January-2016) from the IntAct, BioGRID, InnateDB, InnateDB-all, InnateDB-IMEx and MINT databases by means of the PSICQUIC platform (<http://www.ebi.ac.uk/Tools/webservices/psicquic/view/main.xhtml>) developed by the IMEx consortium. Raw PPI data were processed as previously described. Briefly, protein IDs were converted to Swiss-Prot and Entrez gene ID; TrEMBL, non-protein interactors (e.g. chemicals), obsolete Entrez and Entrez matching to multiple Swiss-Prot identifiers were removed. Raw PPI annotations from different databases were finally merged into a single file for each seed.

**Construction of the PPI network**

The detailed pipeline is described in [9]. Briefly, all PPIs underwent quality control (QC) and filtering. Particularly: i) all non-human taxid annotations were removed; ii) all annotations with poor quality control were removed (i.e. multiple or absent PubMed identifiers, no description of Interaction Detection Method); iii) all proteins whose entire transcript was not reported in brain (<http://www.braineac.org/>) were removed. After interaction detection method reassignment (to pool together similar methodologies) the interactions were then scored taking into consideration the following parameters: i) the number of different publications reporting the interaction (publication score, PS); ii) the number of different methods reporting the interaction (method score, MS); iii) the CrapOme (Contaminant Repository for Affinity Purification) score (CS) (for the first layer only). After computation of the final score (PS+MS+CS), all the interactors with a final score ≤ 2 were discarded because the interaction did not meet our criteria (i – iii, see above). Of note, polyubiquitin-C (UBC), polyubiquitin-B (UBB) and ubiquitin D (UBD) were discarded from the network as they may indicate unspecific binding of ubiquitin to proteins tagged for degradation.

**Topological analysis**

We calculated the inter-interactome degree (IID) for each single node in the network by calculating the number of different interactomes that node belonged to. For each single node, the interactome connection degree (ICD) equates to the IID divided by the number of interactomes (input seeds) in the network and ranges between IDC = 1 (nodes able to bridge all the interactomes in the network) and IDC = 1/number of seeds (nodes unable to bridge any interactomes in the network). Nodes with IDC ≥ 0.6 are inter-interactomes hubs (IIHs).

**Functional Enrichment Analysis and Replication**

We performed Gene Ontology (GO) terms enrichment analyses in g:Profiler (g:GOSt, <http://biit.cs.ut.ee/gprofiler/>) [10] during October-November 2016 using Ensembl v85/Ensembl Genomes v32/33. g:Profiler settings were as follows: enrichment for GO terms biological processes (BP) only; Fisher's one-tailed test as statistical method for enrichment, SCS-threshold as multiple testing correction; statistical domain size was only annotated genes; no hierarchical filtering was included. The following proteins were excluded from analysis because not identified by g:Profiler: ECM29, LINC00312 and LPHN1. Enriched GO-BP terms were grouped into custom-made “semantic classes” (Supplementary File 1). Generic terms (within semantic classes such as Enzyme, General, Metabolism, Motility, Muscle, Physiology, Protein Modification and Virus) were discarded because unspecific. To replicate the enrichment analysis results obtained from g:Profiler, we used PANTHER, an alternative online tool (accessed on the 20^th^ June 2017 and that uses the overrepresentation test as statistical method for enrichment and Bonferroni as multiple testing correction [11], version 11.1 released on 20^th^ December 2016).

Similar semantic classes were grouped into hierarchical groups called ‘functional blocks’ (Supplementary file 1).

**Gene Prioritization – GWAS**

We selected thirty-two relevant PD-SNPs (rs35749011, rs823118, rs10797576, rs6430538, rs1474055, rs115185635, rs12637471, rs34311866, rs11724635, rs6812193, rs356182, rs9275326, rs199347, rs117896735, rs3793947, rs329648, rs76904798, rs11060180, rs11158026, rs1555399, rs2414739, rs14235, rs17649553, rs12456492, rs62120679, rs8118008, rs34016896, rs591323, rs60298754, rs7077361, rs11868035, and rs2823357) [12] including any SNP that was significant in the discovery phase and/or joint analysis. SNPs were elaborated to match the SNP coordinates on the human genome build GRCh37/hg19 (January 2017, <https://data.broadinstitute.org/mpg/snpsnap/>) and retrieve the IDs of the genes contained in the matched loci (reference EU 1000G; locus definition by linkage disequilibrium (LD) r^2^ > 0.5; SNPs of the HLA-locus were included). The genes identified through SNPSNAP were matched with the genes encoding proteins highlighted by WPPINA as relevant to PD associated risk-processes to aid prioritization of genes within the PD-GWAS loci. Results were statistically evaluated by generating 100,000 random gene-sets of similar size to the lists of open reading frames in LD with the PD-GWAS SNPs as detailed below.

**Statistical validation of the GWAS candidate genes prioritization**

To statistically validate our results we generated (100,000 times) two random gene-sets (length of the random gene-sets: n=127 and 83) to numerically represent the lists of ORFs defined by building *cis*-haplotypes (with LD r^2^ = 0.5 and 0.8) around the PD-GWAS SNPs. We then matched the random gene-sets with the proteins contributing to the enrichment of the PD-specific functional blocks. Based on these 100,000 simulated experiments we calculated the p-values associated with the experimental analysis showing strong statistical significance (Supplementary Figure 1, p=0.004 for LD r^2^ ≥ 0.5 and p=0.0053 for LD r^2^ ≥ 0.8). Additionally, analytic p-values were generated using the hypergeometric distribution (with the following parameters: 19 or 27 = real matches for LD r^2^ ≥ 0.8 and LD r^2^ ≥ 0.5 respectively, 2978 = all proteins relevant for PD processes, 17113 = all proteins, 83 or 127 = ORFs in the LD r^2^ ≥ 0.8 and LD r^2^ ≥ 0.5 haplotypes respectively), leading to similar results: (p=0.018 for LD r^2^ ≥ 0.5 and p=0.017 for LD r^2^ ≥ 0.8). All this taken together indicates that the matches between the proteins that are key functional factors in the PD protein network and the genes within the PD loci are statistically unlikely to be random. We undertook an additional validation step by assessing the total number of annotations reported in GO for each single gene within the earlier gene-sets (ORFs) – defined by building *cis*-haplotypes around the PD-GWAS SNPs – to verify potential annotation bias (i.e. whether the number of GO annotation for one [or multiple] gene[s] exceeded that of other genes, thus impacting the specificity of the GWAS loci prioritization). As shown in Supplementary Figure 2, the number of annotations per genes in GO does not influence prioritization specificity.

**Cell type expression**

Cell specific expression of the genes prioritized by the PD-GWAS analysis has been evaluated through the RNA expression archive at www.brainrnaseq.org [13]. The individual expression FPKM data have been downloaded for human temporal lobe cortex mature astrocytes, neurons, microglia, oligodendrocytes and endothelial cells from supplementary materials of Zhang et al [13]. For each cell type we calculated the average FPKM across the individual that were used in the study by Zhang et al [13]. We evaluated cell specific expression profile for each single gene our pipeline prioritized for each PD locus. We considered as enriched any expression value above 5% the average expression across the different cell types (see above).

**Software**

Data was handled, filtered and scored through *in-house* R scripts (<https://www.r-project.org/>) as described before [9]. The final network was visualized through the freely available Cytoscape 2.8.2 software[14][32][32] and analyzed through the network analysis plug-in [14].

**Supplementary references**

1. Siuda J, Fujioka S, Wszolek ZK: **Parkinsonian syndrome in familial frontotemporal dementia**. *Parkinsonism Relat Disord* 2014, **20**(9):957-964.

2. Guidubaldi A, Piano C, Santorelli FM, Silvestri G, Petracca M, Tessa A, Bentivoglio AR: **Novel mutations in SPG11 cause hereditary spastic paraplegia associated with early-onset levodopa-responsive Parkinsonism**. *Mov Disord* 2011, **26**(3):553-556.

3. Kruer MC, Salih MA, Mooney C, Alzahrani J, Elmalik SA, Kabiraj MM, Khan AO, Paudel R, Houlden H, Azzedine H *et al*: **C19orf12 mutation leads to a pallido-pyramidal syndrome**. *Gene* 2014, **537**(2):352-356.

4. Lu CS, Chang HC, Kuo PC, Liu YL, Wu WS, Weng YH, Yen TC, Chou YH: **The parkinsonian phenotype of spinocerebellar ataxia type 3 in a Taiwanese family**. *Parkinsonism Relat Disord* 2004, **10**(6):369-373.

5. Lu CS, Wu Chou YH, Kuo PC, Chang HC, Weng YH: **The parkinsonian phenotype of spinocerebellar ataxia type 2**. *Arch Neurol* 2004, **61**(1):35-38.

6. Puschmann A: **Monogenic Parkinson's disease and parkinsonism: clinical phenotypes and frequencies of known mutations**. *Parkinsonism Relat Disord* 2013, **19**(4):407-415.

7. Schneider SA, Bhatia KP: **Rare causes of dystonia parkinsonism**. *Curr Neurol Neurosci Rep* 2010, **10**(6):431-439.

8. Seo JH, Song SK, Lee PH: **A Novel PANK2 Mutation in a Patient with Atypical Pantothenate-Kinase-Associated Neurodegeneration Presenting with Adult-Onset Parkinsonism**. *J Clin Neurol* 2009, **5**(4):192-194.

9. Ferrari R, Lovering RC, Hardy J, Lewis PA, Manzoni C: **Weighted Protein Interaction Network Analysis of Frontotemporal Dementia**. *J Proteome Res* 2017, **16**(2):999-1013.

10. Reimand J, Arak T, Adler P, Kolberg L, Reisberg S, Peterson H, Vilo J: **g:Profiler-a web server for functional interpretation of gene lists (2016 update)**. *Nucleic Acids Res* 2016, **44**(W1):W83-89.

11. Mi H, Huang X, Muruganujan A, Tang H, Mills C, Kang D, Thomas PD: **PANTHER version 11: expanded annotation data from Gene Ontology and Reactome pathways, and data analysis tool enhancements**. *Nucleic Acids Res* 2017, **45**(D1):D183-D189.

12. Nalls MA, Pankratz N, Lill CM, Do CB, Hernandez DG, Saad M, DeStefano AL, Kara E, Bras J, Sharma M *et al*: **Large-scale meta-analysis of genome-wide association data identifies six new risk loci for Parkinson's disease**. *Nat Genet* 2014, **46**(9):989-993.

13. Zhang Y, Sloan SA, Clarke LE, Caneda C, Plaza CA, Blumenthal PD, Vogel H, Steinberg GK, Edwards MS, Li G *et al*: **Purification and Characterization of Progenitor and Mature Human Astrocytes Reveals Transcriptional and Functional Differences with Mouse**. *Neuron* 2016, **89**(1):37-53.

14. Shannon P, Markiel A, Ozier O, Baliga NS, Wang JT, Ramage D, Amin N, Schwikowski B, Ideker T: **Cytoscape: a software environment for integrated models of biomolecular interaction networks**. *Genome Res* 2003, **13**(11):2498-2504.
