# Supplementary figures and images for: Stratification of candidate genes for Parkinson’s disease using weighted protein-protein interaction network analysis
Source: BMC Genomics. 2018 Jun 13;19:452. doi: 10.1186/s12864-018-4804-9 (PMC6000968; doi:10.1186/s12864-018-4804-9)

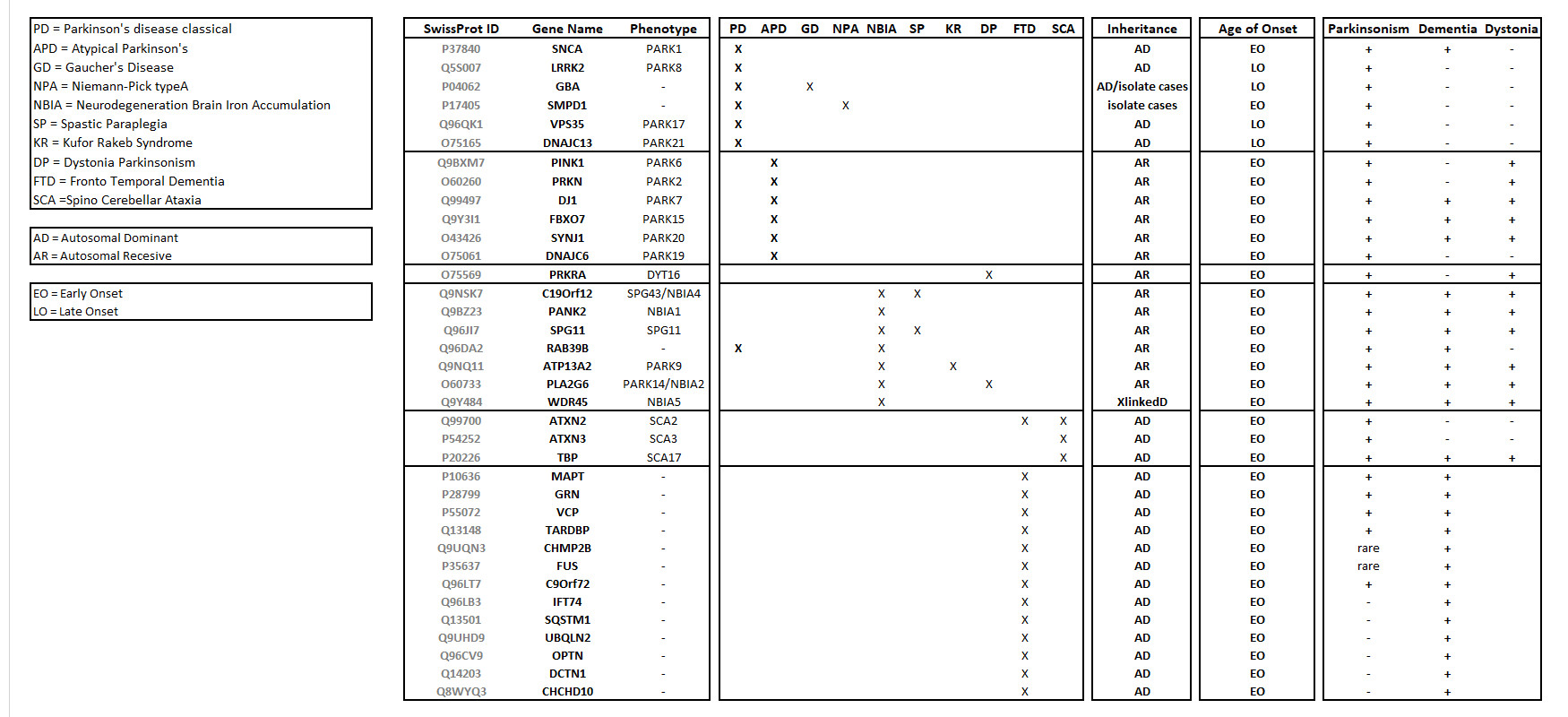

Supplement: Supplementary file 2 — Seeds and Parkinsonian syndromes. (JPG 266 kb) [file 12864_2018_4804_MOESM2_ESM.jpg]

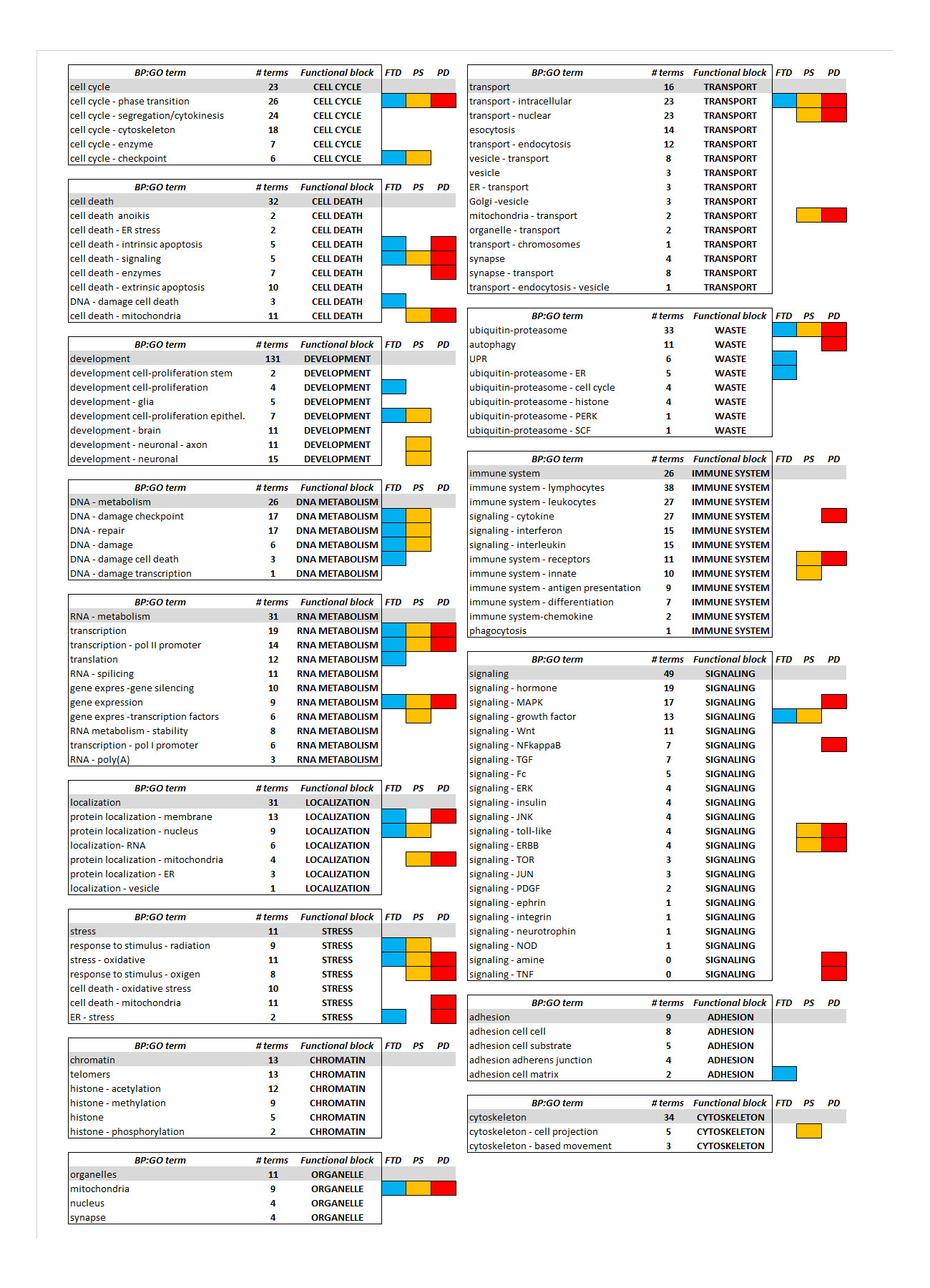

Supplement: Supplementary file 9 — Enrichment Analysis. The biological process-Gene Ontology (BP-GO) terms obtained from the functional enrichment analysis of the entire network (Fig. 1d) were first grouped into semantic classes by semantic similarity and then into functional blocks. More general terms within the same functional blocks are highlighted in grey. Enrichment analysis was then performed for the PD, FTD and PS IIHs separately: enriched semantic classes from these analyses are highlighted in blue for FTD, orange for PS and red for PD. (JPG 790 kb) [file 12864_2018_4804_MOESM9_ESM.jpg]

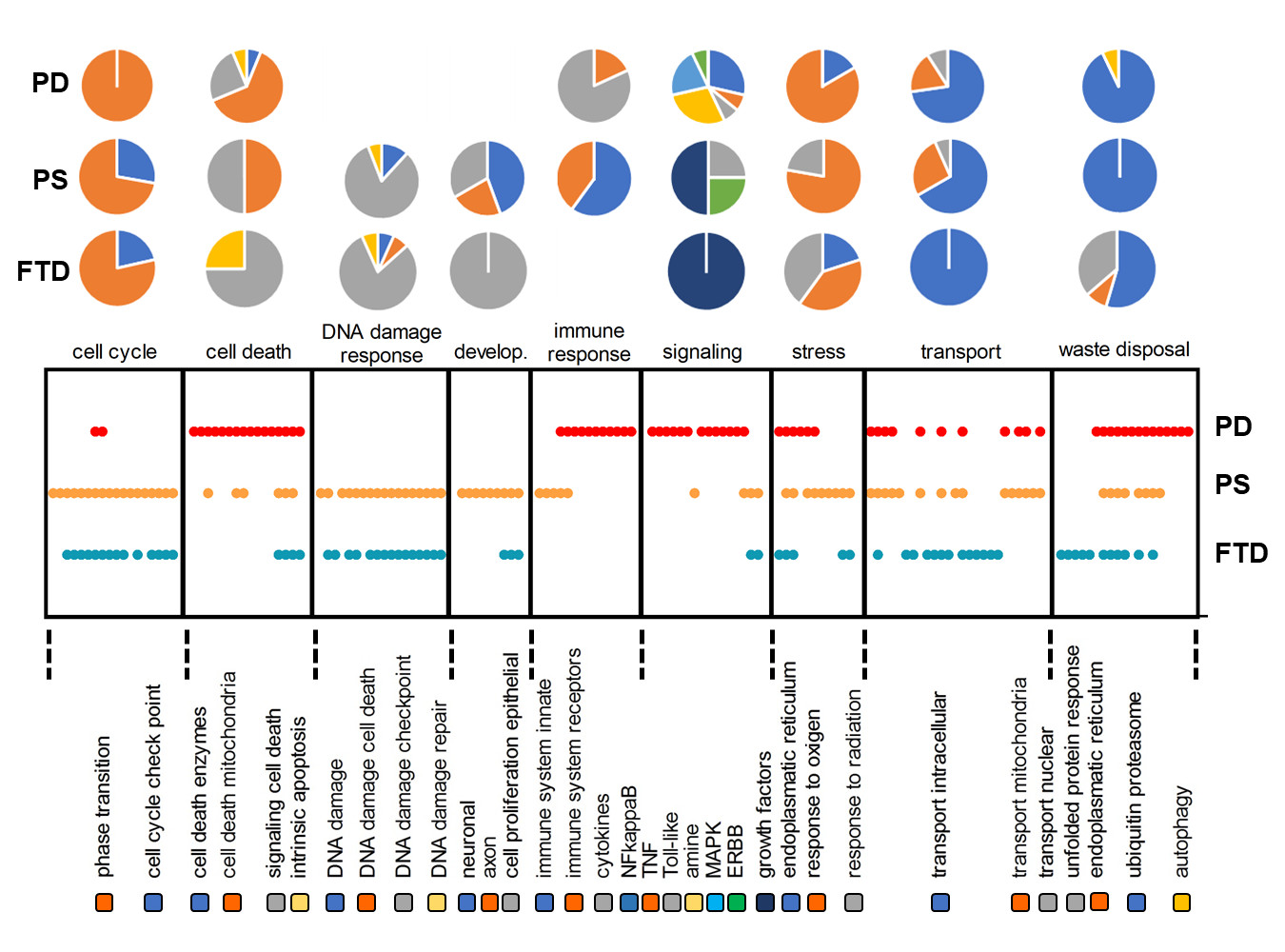

Supplement: Supplementary file 10 — Functional blocks supporting syndrome-specific coherent patterns for PD, FTD and PS. Each semantic class is represented at the bottom of the x axis. Each functional block is found at the top of the x axis. The contribution of each syndrome to each semantic class is defined by colour-coded dots representing single GO terms within each semantic class (PD: red dots, FTD blue dots and PS: orange dots). The pie charts represent the relative distribution of different semantic classes per functional block for either syndrome. (JPG 228 kb) [file 12864_2018_4804_MOESM10_ESM.jpg]

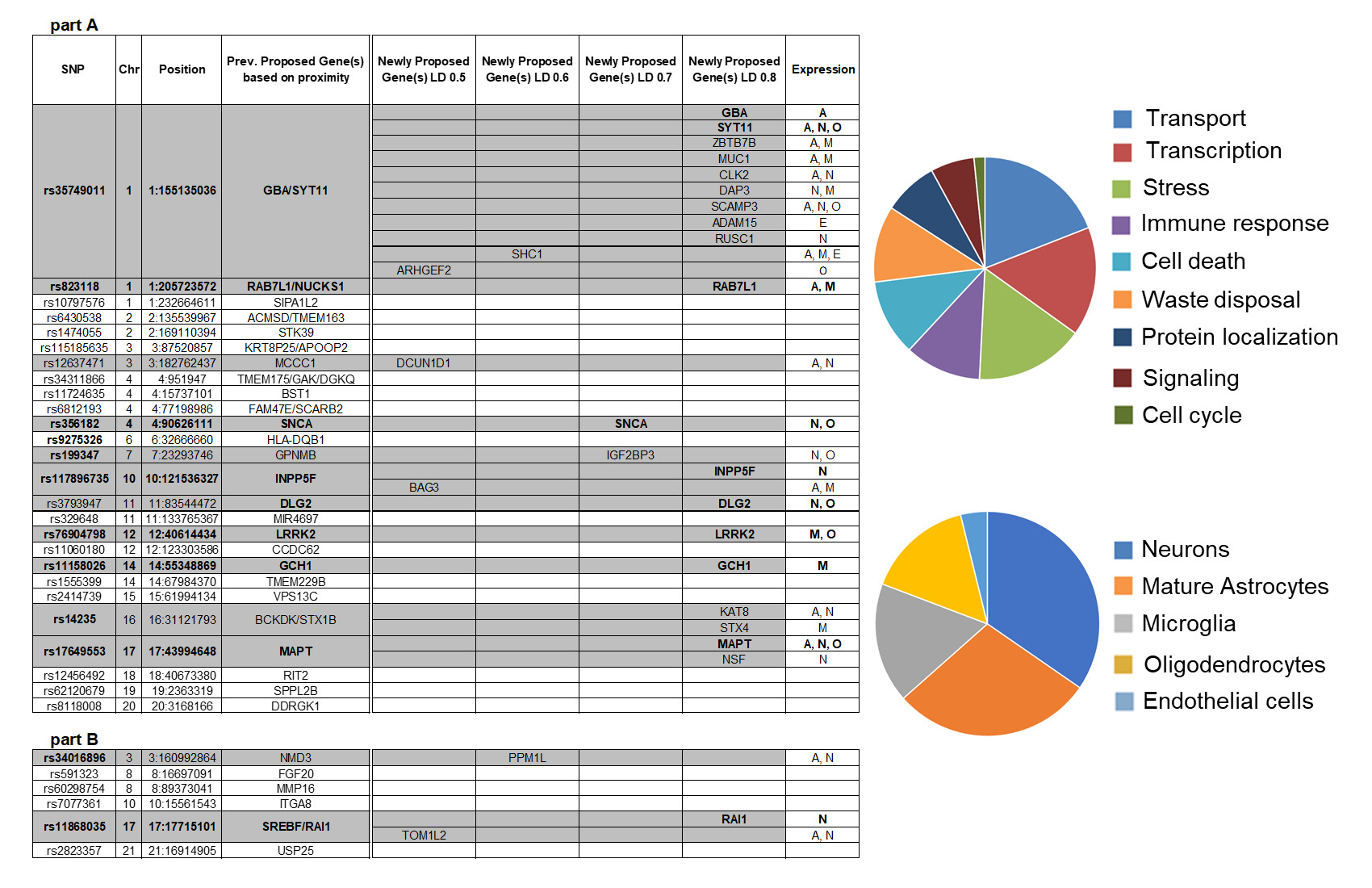

Supplement: Supplementary file 15 — PD-GWAS gene prioritization. Significant SNPs from PD-GWAS and the number of ORFs in LD (from r2 > 0.5 to r2 > 0.8) are shown. Part A contains significant SNPs as per joint analysis, part B contains significant SNPs as per discovery phase. The candidate genes based on proximity are summarized as suggested in the original GWAS. Newly proposed candidate genes within each are identified on the basis of our analysis of the functionally relevant proteins in the PPI network. Genes previously selected by proximity and now also confirmed by functional analysis of the PD-network are in bold font. The top pie chart represents the distribution of proteins across the different relevant processes. In the final column the cell type with major expression (> 5% of average expression) is reported as calculated from the dataset generated by Zhang et al. [13] (A = mature astrocytes, N = neurons, M = microglia, O = oligodendrocytes and, E = endothelial cells). The bottom pie chart represents the distribution of proteins based on cell type expression in human temporal lobe cortex. (JPG 349 kb) [file 12864_2018_4804_MOESM15_ESM.jpg]

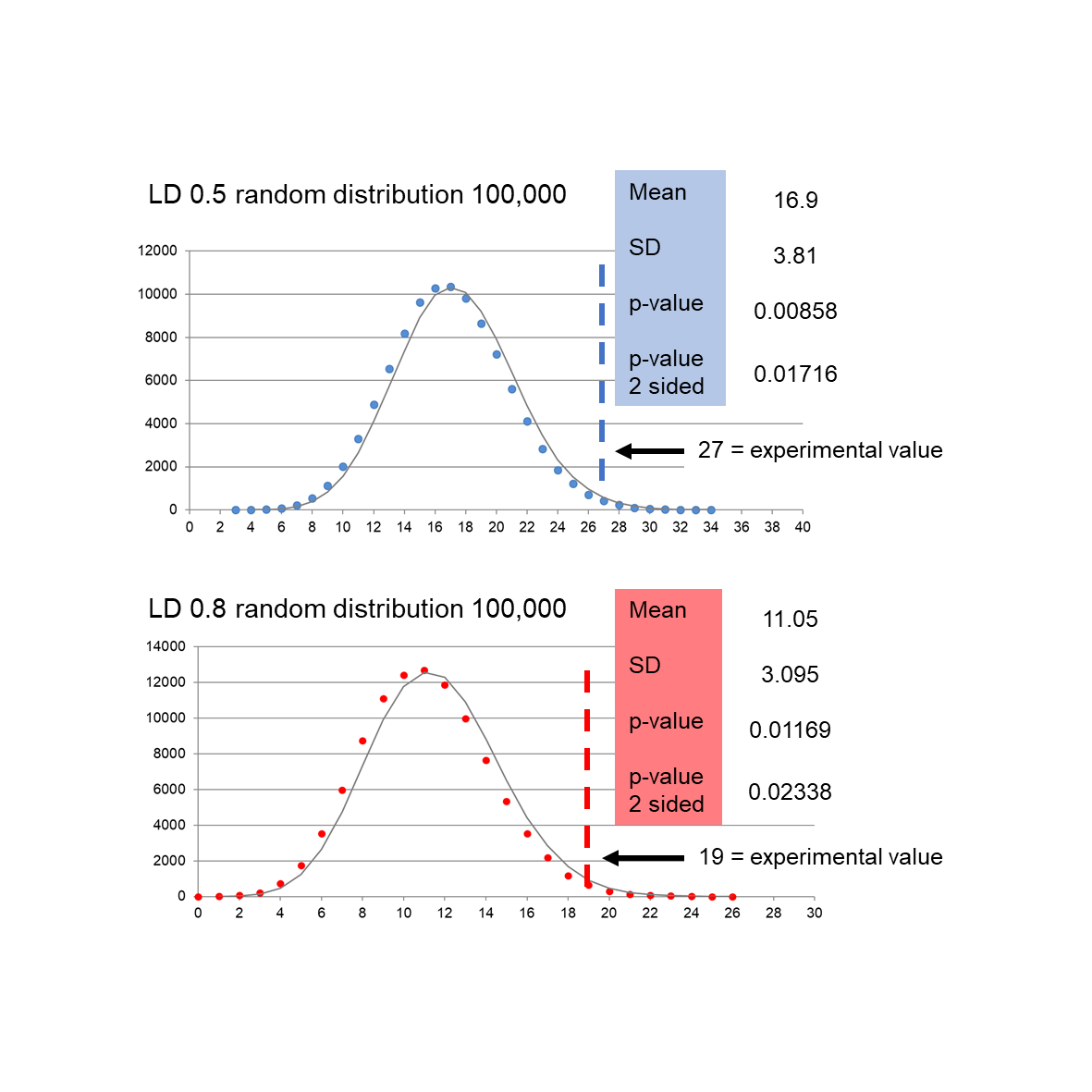

Supplement: Supplementary file 17 — .GO Annotation Frequency. Proteins whose ORF is in the LD blocks around the prioritized SNPs in the PD-GWAS have been evaluate in terms of numbers of GO annotations present in GO for that specific ORF. In red are reported the proteins that correspond to genes that we prioritized with our analysis. In grey, all the other genes are reported. For some of the loci (A) the genes we prioritized was the gene with the maximum number of GO annotations for that locus; in some other cases (B) the genes we prioritized was NOT the gene with the maximum number of GO annotations for that locus. Finally, there are also mixed cases (C). (TIFF 315 kb) [file 12864_2018_4804_MOESM17_ESM.tiff]

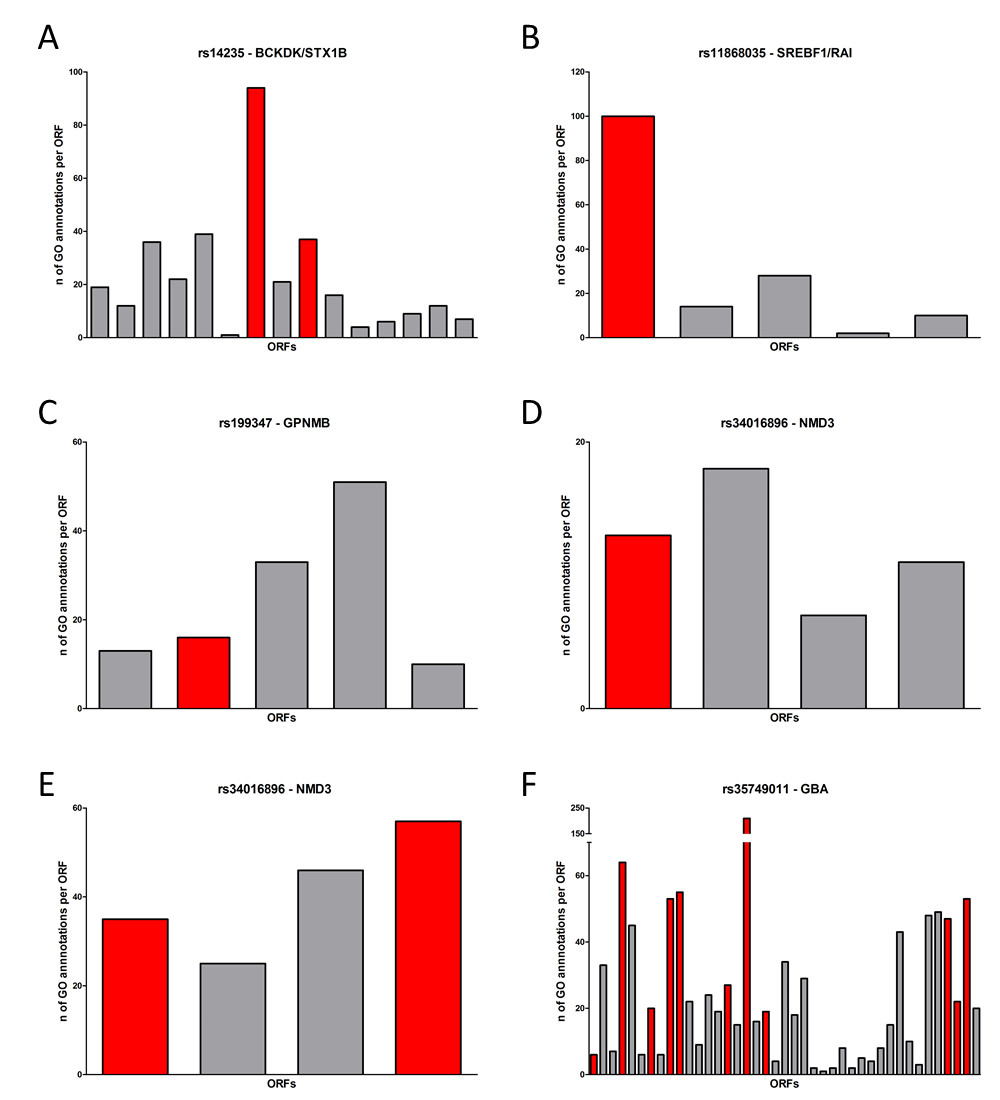

Supplement: Supplementary file 18 — Second layer network topological properties. (JPG 94 kb) [file 12864_2018_4804_MOESM18_ESM.jpg]
