# Supplementary material for: Stratification of candidate genes for Parkinson’s disease using weighted protein-protein interaction network analysis
Source: BMC Genomics. 2018 Jun 13;19:452. doi: 10.1186/s12864-018-4804-9 (PMC6000968; doi:10.1186/s12864-018-4804-9)
Supplement: Supplementary file 3 — Random Distribution. Distribution of the number of matches obtained in 100,000 simulated experiments in which we matched the relevant PD, process-specific, network proteins to randomly generated gene-sets of the same length as the list of ORFs in LD blocks with the top SNPS in the PD-GWAS. The distribution in blue is generated for random gene-sets of the same length as the list of ORFs in LD r2 ≥ 0.5; the distribution in red is generated for random gene-sets of the same length as the list of ORFs in LD r2 ≥ 0.8. (PDF 36 kb) [file 12864_2018_4804_MOESM3_ESM.pdf]

Network	Average n Neighbours	Connection Density	Characteristic Path Length
I layer	2.344	1.2	4.141
I and II layers	5.818	3.4	3.614
