# Supplementary material for: Stratification of candidate genes for Parkinson’s disease using weighted protein-protein interaction network analysis
Source: BMC Genomics. 2018 Jun 13;19:452. doi: 10.1186/s12864-018-4804-9 (PMC6000968; doi:10.1186/s12864-018-4804-9)
Supplement: Supplementary file 4 — Features of the PD, FTD and PS networks. A. Summary of the main features of each interactome and level of overlap across interactomes and inter-interactome hubs (IIH). B. IDC, degree of interactome connection. (PDF 259 kb) [file 12864_2018_4804_MOESM4_ESM.pdf]

A

PD

				Nodes I		IIHs	
Seeds	Nodes I	Nodes I+II	IIHs	∩ PS	∩ FTD	∩ PS	∩ FTD
13	286	4089	24	51 (17.8%)	47 (16.4%)	9 (37.5%)	8 (33.3%)

PS

				Nodes I		IIHs	
Seeds	Nodes I	Nodes I+II	IIHs	∩ PD	∩ FTD	∩ PD	∩ FTD
8	140	2119	25	51 (36.4%)	24 (17.1%)	9 (36%)	11 (44%)

FTD

				Nodes I		IIHs	
Seeds	Nodes I	Nodes I+II	IIHs	∩ PS	∩ PD	∩ PS	∩ PD
12	272	4191	29	24 (8.8%)	47 (17.3%)	11 (37.9%)	8 (27.6%)

B

IDC	PD	IDC	PS	IDC	FTD
0.69	SQSTM1	0.75	ARRB2	0.75	COPS5
0.69	VCP	0.75	EGFR	0.75	EGFR
0.62	APP	0.75	FN1	0.75	ESR1
0.62	BAG2	0.75	HSP90AA1	0.75	FN1
0.62	BIRC2	0.75	USP7	0.75	HSP90AA1
0.62	CUL5	0.63	APP	0.75	HSP90AB1
0.62	EGFR	0.63	AURKA	0.75	HSPA8
0.62	HSP90AA1	0.63	CDK2	0.75	PDCD6IP
0.62	HSP90AB1	0.63	CREBBP	0.75	STUB1
0.62	LRRK2	0.63	ESR1	0.75	TP53
0.62	MDM2	0.63	EZH2	0.75	VCP
0.62	PARK2	0.63	GRB2	0.67	APP
0.62	PSMD2	0.63	GSK3B	0.67	CDK2
0.62	PTGES3	0.63	HIST1H4A	0.67	ELAVL1
0.62	RIPK1	0.63	HSPA8	0.67	EP300
0.62	RNF2	0.63	IKBKE	0.67	FSCN1
0.62	TNFRSF1A	0.63	MDM2	0.67	GNB2L1
0.62	TP53	0.63	PARK2	0.67	HDAC1
0.62	TRAF2	0.63	PML	0.67	HSPA4
0.62	TRAF6	0.63	RNF2	0.67	HTT
0.62	VCAM1	0.63	SP1	0.67	MCM7
0.62	YWHAB	0.63	TP53	0.67	PIN1
0.62	YWHAQ	0.63	UBE2D3	0.67	PML
0.62	YWHAZ	0.63	VCAM1	0.67	RPS3
		0.63	YWHAZ	0.67	TCP1
				0.67	TRIM32
				0.67	TUBA1A
				0.67	VCAM1
				0.67	YWHAZ
